# Mr. Joseph Kershaw

**Published:** 1910-07-30

**Authors:** 


Mr. Joseph Kershaw has died at Chorlton-on-Medlock, Manchester, in his eightieth year. More than half a century ago, in 1857, Mr. Kershaw became an M.R.C.S., and in 1878 he took his licentiate of the Society of Apothecaries.

